# Circulating Liver-Enriched miR-122 in COVID-19 Patients: A Longitudinal Real-Life Study

**DOI:** 10.3390/ijms27052288

**Published:** 2026-02-28

**Authors:** Nicoleta Mihai, Cătălin Tilișcan, Iulia Virginia Iancu, Oana-Alexandra Ganea, Aida-Isabela Adamescu, Ștefan Sorin Aramă, Andreea Letiția Arsene, Simona-Maria Ruță, Victoria Aramă

**Affiliations:** 1 Faculty of Medicine, “Carol Davila” University of Medicine and Pharmacy, 37 Dionisie Lupu Street, 020021 Bucharest, Romania; nicoleta.mihai1@drd.umfcd.ro (N.M.); oana-alexandra.ganea@drd.umfcd.ro (O.-A.G.); simona.ruta@umfcd.ro (S.-M.R.); victoria.arama@umfcd.ro (V.A.); 2 Faculty of Dental Medicine, “Carol Davila” University of Medicine and Pharmacy, 37 Dionisie Lupu Street, 020021 Bucharest, Romania; sorin.arama@umfcd.ro; 3 Faculty of Pharmacy, “Carol Davila” University of Medicine and Pharmacy, 37 Dionisie Lupu Street, 020021 Bucharest, Romania; andreea.arsene@umfcd.ro; 4 “Prof. Dr. Matei Balș” National Institute for Infectious Diseases, 1 Calistrat Grozovici Street, 021105 Bucharest, Romania; 5 “Ştefan S. Nicolau” Institute of Virology, 285 Mihai Bravu Road, 030304 Bucharest, Romania; iulia_iancu2005@yahoo.com

**Keywords:** miR-122, COVID-19, remdesivir, paracetamol, drug-induced liver injury, systemic inflammation

## Abstract

Abnormal liver function tests are frequently reported in patients with COVID-19. This study aimed to identify potential treatment-associated hepatocellular injury in COVID-19 patients by dynamically assessing circulating miR-122, a biomarker with high hepatic specificity and sensitivity. An exploratory approach was additionally used, given the limited evidence regarding factors influencing miR-122 expression in this setting. We performed a prospective cohort study including 96 adult participants enrolled at a tertiary hospital in Bucharest, Romania, between March 2022 and July 2023: 78 COVID-19 patients (57 with baseline and follow-up miR-122 assessment after 5 days of treatment and 21 with a single measurement) and 18 non-COVID-19 participants included for comparison. Plasma miR-122 levels were measured using quantitative polymerase chain reaction, normalized to U6 small nuclear RNA, and expressed as log_10_(2^−ΔCt^). No associations were observed between miR-122 expression and remdesivir administered for standard treatment durations (3–5 days) or other COVID-19–specific therapies. However, a duration-dependent relationship with remdesivir cannot be excluded. Moreover, therapeutic paracetamol use prior to presentation was positively associated with miR-122 expression at follow-up and remained significant after adjustment. Additionally, bivariate analyses revealed inverse correlations between baseline miR-122 and inflammatory biomarkers, with multivariable analysis showing an independent positive association with lymphocyte count.

## 1. Introduction

MicroRNAs are endogenous non-coding ribonucleic acid (RNA) molecules, approximately 19–24 nucleotides in length, which regulate gene expression at the post-transcriptional level, generally exerting a repressive effect through messenger RNA degradation [1,2]. The expression of different microRNAs varies across tissues and according to physiological or pathological processes [1]. In addition, microRNAs are released into the bloodstream, where incorporation into protein complexes or microvesicles confers remarkable stability [3], enabling their quantification and use as biomarkers in a wide range of diseases.

MicroRNA-122 (miR-122) is one of the most abundantly expressed microRNAs in the human body and is almost exclusively of hepatic origin, accounting for approximately 70% of the total microRNA content in the liver [4,5]. It plays a key role in hepatocyte development, differentiation, homeostasis, and regeneration. miR-122 is also an important factor in the replication of hepatotropic viruses, inhibiting hepatitis B virus (HBV) replication while paradoxically promoting hepatitis C virus (HCV) replication. In addition, miR-122 acts as a hepatic tumor suppressor, with reduced expression being associated with the development of hepatocellular carcinoma [2]. In these conditions, miR-122 has emerged as a valuable biomarker with potential therapeutic implications.

Studies have shown that, in the presence of acute liver injury, increases in circulating miR-122 levels precede elevations in serum aminotransferases and may occur even in the absence of biochemical evidence of hepatocellular injury. Moreover, the increase in miR-122 levels is more pronounced than that of aminotransferases and subsequently declines more rapidly, thereby more accurately reflecting the extent of hepatic injury. Finally, unlike miR-122, elevated aminotransferase levels are not liver-specific and may also occur in other conditions, such as muscle injury. Consequently, miR-122 has emerged as a highly sensitive and specific biomarker of liver damage, outperforming conventional aminotransferases, and has therefore been extensively investigated in drug-induced liver injury [1].

In the context of COVID-19, only a limited number of studies have evaluated miR-122 [6,7]. These investigations, conducted predominantly during the early pandemic waves, focused primarily on associations with disease severity and mortality, while other potential clinical applications of this biomarker remain largely unexplored.

Abnormal liver function tests are frequently reported in COVID-19 patients, being described in up to 78% of cases [8,9]. Multiple mechanisms have been proposed to explain liver injury in these patients, including direct viral damage, systemic inflammation, drug-induced liver injury, and HBV reactivation [10,11]. However, the contribution of each mechanism remains incompletely understood. In particular, distinguishing the relative contribution of drug-induced liver injury from disease-related mechanisms has proven challenging, as liver enzyme abnormalities in COVID-19 are multifactorial and difficult to attribute robustly to specific exposures in the absence of longitudinal designs and liver-specific biomarkers.

Our group has focused on investigating treatment-related hepatotoxicity, and in a previous retrospective study we identified an association between certain COVID-19 therapies and elevated liver enzymes [9]. However, conventional biochemical markers lack hepatic specificity and may not reliably detect subclinical hepatocellular injury.

Given the established utility of circulating miR-122 as a highly sensitive and liver-enriched biomarker, particularly in the context of drug-induced liver injury, we sought to leverage its dynamic assessment to more specifically evaluate potential treatment-associated hepatocellular injury in patients with COVID-19. By prospectively monitoring miR-122 levels in a real-life cohort of patients treated according to current clinical practice, this study aims to clarify whether commonly used COVID-19 therapies are associated with hepatocellular injury beyond the effects of the infection itself. To our knowledge, this is the first longitudinal study to evaluate miR-122 expression in relation to COVID-19 therapy.

## 2. Results

### 2.1. Characteristics of the Study Participants

A total of 96 participants were included in the study: 78 patients with confirmed COVID-19 and 18 non-COVID-19 participants. Among the COVID-19 patients, 21 had a single miR-122 measurement, while 57 had longitudinal miR-122 assessment with a second blood sample collected after 5 days of COVID-19 therapy (interquartile range [IQR] 4–6). Baseline characteristics of the study participants, stratified by COVID-19 status and disease severity, are summarized in Supplementary Table S1.

COVID-19–positive patients (*n* = 78) had a median age of 65 years (IQR 47–74), with a female-to-male ratio of 2.4:1, and a body mass index (BMI) of 25.75 kg/m^2^ (IQR 22–29). A majority of patients presented with at least one comorbidity (*n* = 65, 83.3%). The most prevalent comorbid conditions were cardiovascular disease (*n* = 47, 60.3%) and dyslipidemia (*n* = 21, 26.9%). Eleven patients (14.1%) had multiple comorbidities. The median Charlson Comorbidity Index was 2 (IQR 0–4).

All patients were enrolled during the period of Omicron variant circulation. Genetic sequencing was performed in two patients, identifying the original Omicron lineage (B.1.1.529) in a patient who presented in March 2022 and the XBB.1.9.1/FL.2 subvariant in a patient enrolled in May 2023. Vaccination history was available for 66 patients, of whom 47 (71.2%) had been vaccinated against SARS-CoV-2, most commonly with two (*n* = 20, 42.5%) or three doses (*n* = 25, 53.2%) of mRNA-based vaccines.

At the time of the first blood sample collection for miR-122 analysis, the median time from symptom onset was 5 days (IQR 3–7). Most patients had mild (*n* = 38, 39.6%) or moderate COVID-19 (*n* = 32, 33.3%). Eight patients (10.3%) developed severe COVID-19, requiring oxygen therapy with a median flow rate of 4 L/min (IQR 2–13). Only one patient (1.3%) required admission to the intensive care unit. No deaths were recorded. Seventy-one patients (91%) were hospitalized, with a median length of hospital stay of 6 days (IQR 4–8; range 3–49).

Elevated aminotransferase levels at presentation and/or at follow-up were observed in 25 COVID-19 patients (32%), with most elevations classified as grade 1 (less than three times the upper limit of normal). The detailed distribution and severity of abnormalities in liver-related biochemical parameters among the study participants are presented in Supplementary Table S2.

The non-COVID-19 participants (*n* = 18) had a median age of 51 years (IQR 44–58), with a female-to-male ratio of 2:1, and a median BMI of 28 kg/m^2^ (IQR 24.8–31). Seven participants (38.9%) had associated comorbidities, including cardiovascular disease (*n* = 5, 27.8%), dyslipidemia (*n* = 1, 5.6%), and diabetes mellitus (*n* = 1, 5.6%). The median Charlson Comorbidity Index was 0.5 (IQR 0–1).

### 2.2. Treatment Exposure of the Study Participants

Prior to hospital presentation, 67 COVID-19 patients (85.9%) had self-administered paracetamol at home for a median duration of 3 days (IQR 2–4), with median cumulative doses of 2 g (IQR 1–4.5) and a maximum daily dose of 3 g. In addition, 11 patients (14.1%) had taken nonsteroidal anti-inflammatory drugs (NSAIDs), and 16 (20.5%) had used antibiotics.

Among the 57 patients with longitudinal miR-122 assessment, 48 (84.2%) reported prior paracetamol use, with similar patterns of dose and duration as described above. The prevalence of prior NSAIDs and antibiotic use was also comparable. The treatments administered during hospitalization in this subgroup are summarized in Table 1.

A substantial proportion of patients (74.4%) were receiving treatment for underlying comorbidities, most commonly beta-blockers, statins, diuretics, and renin-angiotensin system inhibitors. Detailed distribution of major chronic medications is provided in Supplementary Table S3.

### 2.3. Associations of Baseline miR-122 with Clinical and Laboratory Parameters in COVID-19 Patients

**Figure 1:**
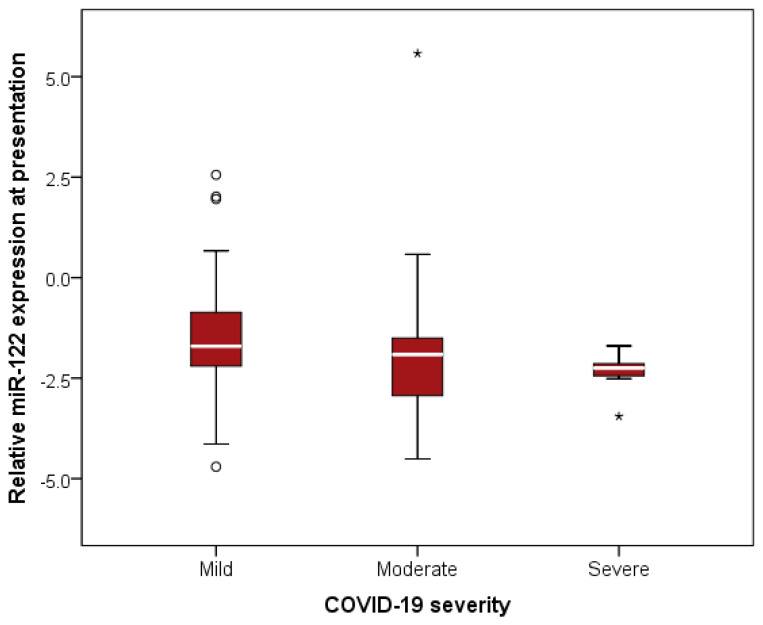
Relative miR-122 expression at presentation across COVID-19 severity groups. Relative miR-122 expression was calculated as log_10_(2^−ΔCt^), where ΔCt represents the difference between miR-122 and U6 cycle threshold (Ct) values. Boxes represent the interquartile range (IQR), with the horizontal line indicating the median. Whiskers extend to 1.5 × IQR. Circles denote moderate outliers (1.5–3 × IQR) and asterisks indicate extreme outliers (>3 × IQR). No statistically significant differences were observed between groups (Kruskal–Wallis test, *p* = 0.291).

In bivariate analysis, relative miR-122 expression at presentation was inversely correlated with COVID-19 severity (Spearman’s ρ = −0.230, *p* = 0.043). However, comparative group analysis using the Kruskal–Wallis test did not reveal statistically significant differences in miR-122 expression across COVID-19 severity categories (Figure 1, Supplementary Table S1). In addition, miR-122 expression was positively correlated with peripheral oxygen saturation (SpO_2_) at presentation and inversely correlated with age, Charlson Comorbidity Index, and chronic treatment with angiotensin receptor blockers (ARBs) (Table 2). miR-122 expression did not differ by sex (*p* = 0.355) and was not associated with BMI (*p* = 0.706) among COVID-19 patients. No significant differences were observed according to COVID-19 vaccination status (*p* = 0.200).

When analyzing miR-122 expression at presentation in relation to inflammatory biomarkers, statistically significant inverse correlations were identified with fibrinogen, C-reactive protein (CRP), and interleukin-6 (IL-6), along with a positive correlation with lymphocyte count. The strongest relationship was noted for lymphocyte count (Spearman’s ρ = 0.399, *p* < 0.001) (Table 2). No significant associations were observed with serum interleukin-1 (IL-1), tumor necrosis factor-α (TNF-α), plasminogen activator inhibitor-1 (PAI-1), ferritin levels, or neutrophil count.

In a multivariable linear regression model including lymphocyte count, age, Charlson Comorbidity Index, SpO_2_ at presentation, and treatment with ARBs, only lymphocyte count remained independently associated with miR-122 expression (B = 0.000241, *p* = 0.040). Given the interrelated nature of inflammatory biomarkers, lymphocyte count was included in the model as a representative marker of systemic inflammation, based on the strength of its association with miR-122, while other inflammatory markers did not show consistent independent associations in adjusted analyses.

In a subgroup analysis of patients with at least 5 days since symptom onset at presentation, stronger correlations were observed between baseline miR-122 expression and most of the clinical and biological parameters mentioned above, including inflammatory markers, with the exception of IL-6, for which the association was no longer statistically significant. Notably, the correlation with lymphocyte count remained among the strongest, while an additional significant association emerged with prior NSAIDs use. These findings are presented in Table 2 alongside those from the overall COVID-19 cohort.

Notably, miR-122 expression did not correlate with liver-related biochemical parameters, including alanine aminotransferase (ALT), aspartate aminotransferase (AST), gamma-glutamyl transferase (GGT), alkaline phosphatase (ALP), lactate dehydrogenase (LDH), or total bilirubin (TBIL) (all *p* > 0.05).

### 2.4. miR-122 Expression After COVID-19 Treatment

**Figure 2:**
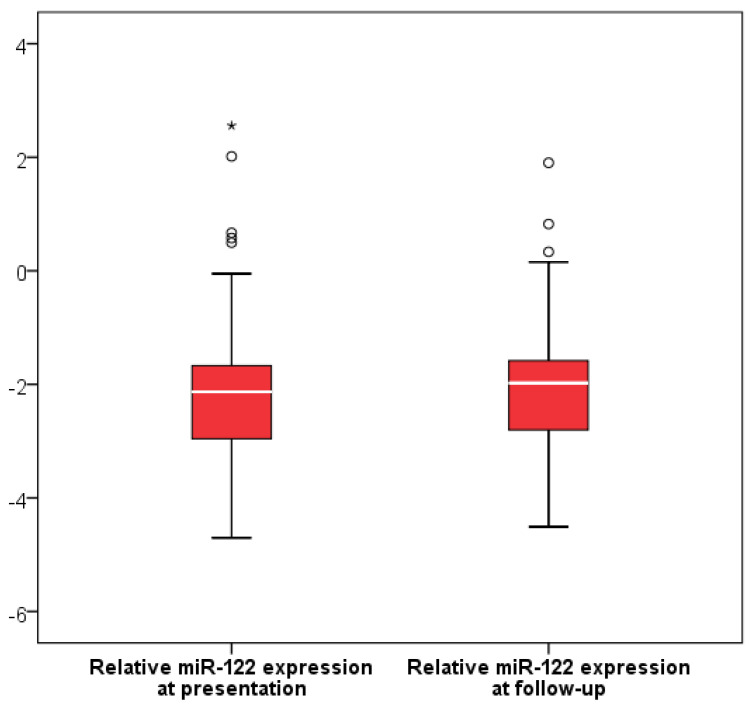
Relative miR-122 expression at baseline and follow-up. Boxes represent the interquartile range (IQR), with the horizontal line indicating the median. Whiskers extend to 1.5 × IQR. Circles denote moderate outliers (1.5–3 × IQR) and asterisks indicate extreme outliers (>3 × IQR). No significant difference was observed between the two time points (Wilcoxon signed-rank test, *p* = 0.718).

**Figure 3:**
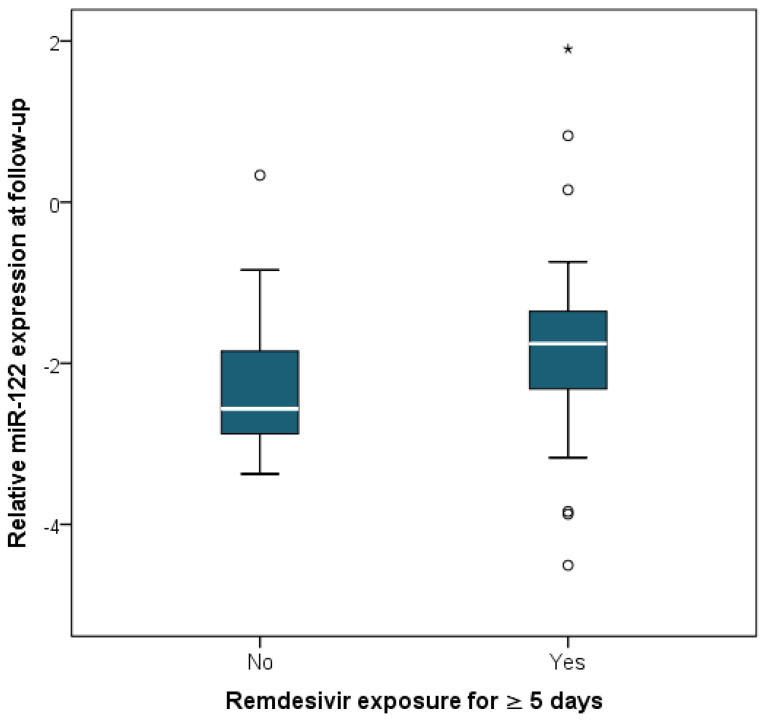
Relative miR-122 expression at follow-up stratified by remdesivir exposure for ≥5 days. Boxes represent the interquartile range (IQR), with the horizontal line indicating the median. Whiskers extend to 1.5 × IQR. Circles denote moderate outliers (1.5–3 × IQR) and asterisks indicate extreme outliers (>3 × IQR). Higher miR-122 levels were observed in patients receiving remdesivir for at least 5 days (Mann–Whitney U test, *p* = 0.012).

**Figure 4:**
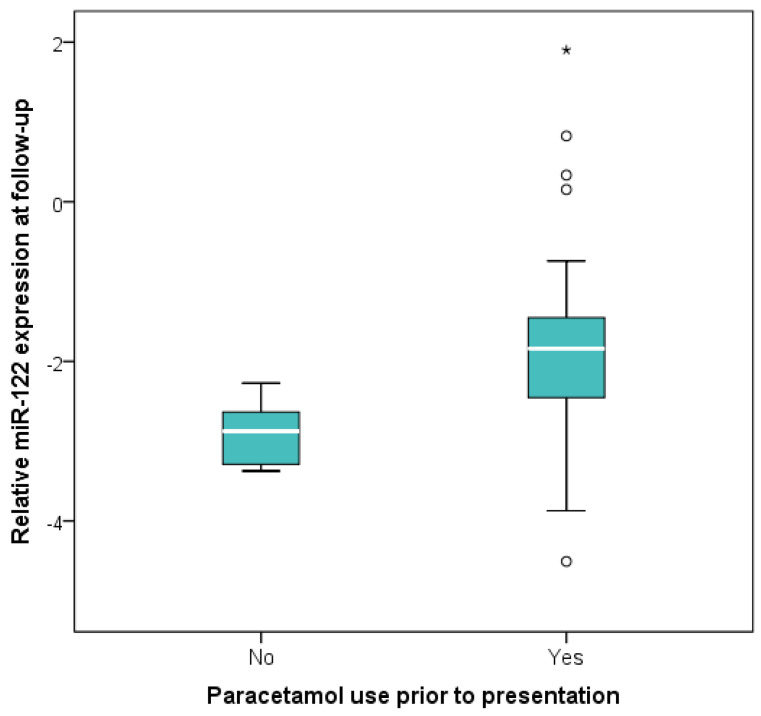
Relative miR-122 expression at follow-up stratified by paracetamol use prior to presentation. Boxes represent the interquartile range (IQR), with the horizontal line indicating the median. Whiskers extend to 1.5 × IQR. Circles denote moderate outliers (1.5–3 × IQR) and asterisks indicate extreme outliers (>3 × IQR). miR-122 levels were significantly higher in patients reporting paracetamol use before presentation (Mann–Whitney U test, *p* = 0.001).

Follow-up measurements were obtained after a median of 5 days of therapy (IQR 4–6) in the subgroup with longitudinal assessment (*n* = 57). No statistically significant differences in relative miR-122 expression were observed between baseline and follow-up levels (Wilcoxon signed-rank test, *p* = 0.718) (Figure 2). However, miR-122 expression at follow-up was higher in patients who received remdesivir for at least 5 days compared with those who did not (*p* = 0.012) (Figure 3), as well as in patients who had received paracetamol prior to presentation (*p* = 0.001) (Figure 4) or who were on chronic beta-blocker (*p* = 0.040) or clopidogrel therapy (*p* = 0.024). In a multivariable linear regression model (R = 0.514, *p* = 0.007) including variables significant in bivariate analysis and adjusted for baseline miR-122 levels, only paracetamol administration prior to presentation (B = 1.027, β = 0.337, *p* = 0.011) remained independently associated with relative miR-122 expression at follow-up.

No significant associations were observed between miR-122 expression at follow-up and other COVID-19–specific therapies (including low-molecular-weight heparin, favipiravir, molnupiravir, tocilizumab, monoclonal antibodies, corticosteroids), nor with antibiotic therapy (all *p* > 0.05).

Relative miR-122 expression at follow-up was not significantly associated with ALT, AST, GGT, or other liver-related biochemical parameters, nor with inflammatory biomarkers or other routine laboratory tests measured at the same time point (all *p* > 0.05).

Changes in routine laboratory parameters between baseline and follow-up are presented in Supplementary Table S4. Notably, lymphocyte counts were significantly higher at follow-up, while other inflammatory markers, including CRP and fibrinogen, were lower. In contrast, liver-related biochemical parameters showed no significant variation between baseline and follow-up.

### 2.5. Comparison of Relative miR-122 Expression Between COVID-19 and Non-COVID-19 Participants

**Figure 5:**
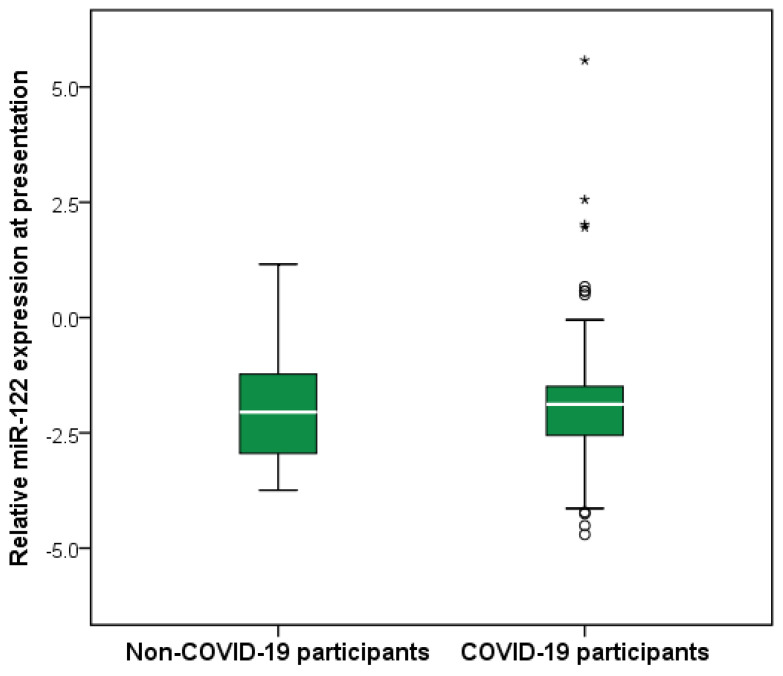
Comparison of relative miR-122 expression between COVID-19 and non-COVID-19 participants. Boxes represent the interquartile range (IQR), with the horizontal line indicating the median. Whiskers extend to 1.5 × IQR. Circles denote moderate outliers (1.5–3 × IQR) and asterisks indicate extreme outliers (>3 × IQR). No significant difference was observed between groups (Mann–Whitney U test, *p* = 0.978).

Relative miR-122 expression at presentation did not differ significantly between COVID-19 and non-COVID-19 participants (Mann–Whitney U test, *p* = 0.978). Median values and interquartile ranges were comparable, with largely overlapping distributions between the two groups (Figure 5).

## 3. Discussion

Clinical experience and published evidence indicate a high prevalence of aminotransferase elevations among COVID-19 patients, with treatment-associated hepatotoxicity being suggested as a contributing factor. Our previous observations supported this hypothesis, highlighting associations between certain therapeutic regimens and liver enzyme elevations [9]. On this basis, the present study aimed to further explore this potential relationship through assessment of miR-122, a biomarker with high hepatic specificity and sensitivity for detecting acute hepatocellular injury. Unlike conventional biochemical markers, circulating miR-122 may capture early or subtle hepatocellular stress that does not necessarily translate into detectable aminotransferase elevations, making it particularly suitable for investigating treatment-related liver effects in multifactorial clinical settings such as COVID-19. In addition, an exploratory approach was used, motivated by the limited evidence regarding factors influencing circulating miR-122 levels in COVID-19 patients, thereby allowing for the identification of potentially novel associations.

### 3.1. miR-122 Expression and COVID-19-Related Treatments

Several therapeutic agents commonly used during the COVID-19 pandemic have been associated with elevations in liver enzymes, although the strength and consistency of this association vary across drug classes. Remdesivir has been linked to increases in aminotransferase levels in both clinical trials [12] and real-world observational studies [13], although other analyses have not demonstrated an excess risk compared with control groups [14]. Favipiravir has also been associated with mild to severe liver enzyme elevations in observational studies [15]. Tocilizumab has been reported to cause liver function test abnormalities in both rheumatologic and COVID-19 clinical settings [16,17]. Transient elevations in aminotransferases have additionally been described during therapy with low-molecular-weight heparins [18], commonly used in COVID-19 for thromboembolic prophylaxis. In contrast, newer antivirals such as molnupiravir have generally demonstrated favorable hepatic safety profiles in clinical trials [19] and real-world analyses [20], although overall clinical experience remains limited. Corticosteroids, widely used in COVID-19 management, have not been linked to hepatotoxicity at the doses and treatment durations currently recommended in this setting, although rare cases of liver injury have been described with prolonged or high-dose exposure in other clinical contexts [21]. Overall, interpretation of these findings remains challenging, as most studies have relied on conventional biochemical markers, and attribution of liver injury to individual agents is complicated by disease severity, systemic inflammation, hypoxia, and the frequent concomitant use of multiple potentially hepatotoxic medications.

To provide a more sensitive assessment of potential treatment-related hepatocellular injury, we used a liver-specific molecular biomarker. Circulating miR-122 levels remained comparable between baseline and follow-up, suggesting that COVID-19 treatment regimens administered during hospitalization were not associated with detectable hepatocellular injury.

Nevertheless, a possible relationship between remdesivir administration for at least 5 days and higher follow-up miR-122 levels was initially observed in our cohort, although this was no longer evident after accounting for baseline miR-122 expression and concomitant treatment exposures. This finding is consistent with our previous study, which did not identify an association between remdesivir use and liver enzyme elevations [9]. At the same time, our initial observation is in line with data from phase I trials of remdesivir, in which ALT elevations were more frequently reported at higher doses and/or with longer treatment durations in healthy volunteers [12]. Therefore, while our data together with most available clinical evidence suggest that, at the treatment durations commonly used in clinical practice (3–5 days), remdesivir is not associated with changes indicative of hepatocellular injury, further investigation of miR-122 dynamics in patients with prolonged exposure remains justified in order to clarify a possible duration-dependent effect.

More consistently, prior paracetamol use was associated with higher miR-122 expression at follow-up in our cohort, and this relationship persisted after adjustment for potential confounders. These findings suggest that even therapeutic, subtoxic doses of paracetamol may influence liver function, possibly by rendering hepatocytes more susceptible to injury and through cumulative effects with other COVID-19-related factors. This interpretation is supported by experimental data indicating that exposure to low doses of paracetamol can induce hepatic stress. In a study by Jetten et al. conducted in healthy volunteers, low-dose paracetamol administration resulted in increased expression of specific microRNAs (miR-19b and miR-29c) along with activation of immune and oxidative stress–related pathways resembling early responses to toxic exposure [22]. However, no increase in miR-122 levels was detected, supporting the idea that alterations in miR-122 expression depend on the broader pathological context rather than on paracetamol exposure alone.

### 3.2. miR-122 Expression and Treatments for Comorbid Conditions

With regard to treatments for comorbid conditions, some associations were observed between baseline miR-122 expression and chronic use of ARBs, as well as between follow-up miR-122 levels and chronic use of beta-blockers or clopidogrel. However, the lack of persistence after adjustment suggests that these findings probably reflect confounding by underlying patient characteristics and overall clinical context rather than independent drug-related effects on miR-122 expression. Notably, miR-122 expression was not associated with chronic statin therapy. This finding is consistent with current data suggesting that mild aminotransferase elevations observed during statin treatment are typically transient and not considered indicative of hepatic injury [23].

### 3.3. miR-122 Expression and Liver-Related Biochemical Parameters

In our study, miR-122 expression was not correlated with aminotransferase levels or other liver-related biochemical parameters. Similar findings have been reported in patients with chronic HCV infection [24]. In contrast, other studies have described correlations between miR-122 and aminotransferases in both COVID-19 patients [6] and in other clinical settings, including paracetamol-induced acute liver injury [25] and acute respiratory distress syndrome (ARDS) [3]. Notably, at least some of these studies included patients with severe hepatic injury. In this context, concordance between miR-122 and aminotransferase levels is expected, given their concurrent massive release from hepatocytes. By contrast, in settings of mild or subclinical liver damage, a more sensitive biomarker such as miR-122 may exhibit changes independent of aminotransferases, which may explain the lack of correlation observed in our cohort.

### 3.4. miR-122 Expression and Systemic Inflammation

In line with published data, baseline miR-122 expression in our study showed inverse associations with several inflammatory biomarkers, namely fibrinogen, CRP, and IL-6, although these were not retained in adjusted analyses. Instead, the positive correlation with lymphocyte count remained independently associated with miR-122 expression, suggesting that this parameter may capture a distinct dimension of the host immune response, integrating both inflammatory burden and immune competence, and is less susceptible to collinearity with other inflammatory biomarkers. To our knowledge, the relationship between miR-122 levels and lymphocyte count has not previously been investigated in COVID-19 patients, although their association has been described in other pathological contexts, such as chronic HBV infection [26]. The other inflammatory biomarkers assessed in our cohort, including IL-1, TNF-α, PAI-1, ferritin, and neutrophil count, did not appear to be related to miR-122 expression.

Interestingly, in patients presenting later in the disease course, corresponding to the hyperinflammatory phase of COVID-19, the associations between miR-122 and inflammatory markers appeared stronger, and a direct relationship with prior NSAIDs exposure also emerged. By contrast, at follow-up, when inflammatory markers had decreased significantly compared with baseline, miR-122 expression no longer correlated with their levels.

Our observations are consistent with findings reported by Gutmann et al., who described an inverse correlation between circulating miR-122 and CRP levels in COVID-19 patients [6]. Furthermore, substantial evidence from multiple experimental and pathological contexts indicates that pro-inflammatory stimuli or systemic inflammation lead to a reduction in miR-122 expression [27,28]. This likely reflects a functional adaptation of the liver during inflammatory stress, with its activity shifting from basal metabolic roles toward acute-phase response and host defense mechanisms [29]. In this setting, downregulation of miR-122 may enable derepression of genes necessary for proinflammatory pathways [30].

However, data from the literature are inconsistent, with some studies reporting positive correlations between miR-122 and inflammatory biomarkers such as CRP, IL-6, or TNF-α—particularly in patients with prediabetes or type 2 diabetes mellitus [31,32]—whereas others have found no significant associations in patients with ARDS [3]. One plausible explanation for these differences is that circulating miR-122 levels are influenced by multiple, potentially opposing mechanisms. For instance, concurrent acute hepatocellular injury may lead to increased circulating miR-122 levels due to passive release from damaged hepatocytes, which could mask the effects of inflammation on miR-122 expression.

Although in our cohort most associations with inflammatory biomarkers were not retained after adjustment, the consistency in their direction, concordance with existing literature, and variation according to disease stage, together with the persistent association with lymphocyte count, support the biological relevance of an inverse relationship between miR-122 expression and systemic inflammation.

### 3.5. miR-122 Expression and COVID-19 Severity

An inverse relationship between baseline miR-122 expression and COVID-19 severity was suggested in our initial analyses, but this pattern did not translate into clear differences across severity groups. Given the inverse correlations between miR-122 and inflammatory biomarkers, this finding may reflect the close link between disease severity and systemic inflammation, rather than an independent effect of COVID-19 severity on miR-122 expression. By contrast, in a cohort enrolled during the early pandemic waves, Gutmann et al. reported higher miR-122 levels in patients with severe disease compared with those with mild forms [6]. The differing results may be explained by the larger proportion of critically ill patients included in their study, in whom hepatocellular injury is likely more pronounced and may therefore represent a dominant determinant of circulating miR-122 levels.

### 3.6. miR-122 Expression in COVID-19 Patients Versus Non-COVID-19 Participants

Several studies have shown that circulating microRNA profiles may have a diagnostic role in COVID-19, with multiple microRNAs reported to be highly upregulated in infected patients [33,34]. The involvement of these microRNAs—including miR-122—in host–SARS-CoV-2 interactions has also been discussed [35]. In a recent study published in 2024, Franco et al. reported significantly higher miR-122 expression in 40 COVID-19 patients compared with healthy volunteers. These patients were enrolled between March and May 2020, and most had severe disease [7]. By contrast, Akula et al. observed significantly lower miR-122 expression in 12 patients with moderate-to-severe COVID-19, enrolled in 2020, when compared with healthy controls [36]. The divergent findings may be explained by the same mechanism discussed above, namely the interplay between systemic inflammation and hepatocellular injury, which influences circulating miR-122 levels. This variability is also consistent with reports describing a heterogeneous plasma proteomic response in patients with severe COVID-19 [37]. In our cohort, miR-122 expression levels were comparable between COVID-19 patients and uninfected participants. This finding suggests that SARS-CoV-2 infection alone may not be sufficient to induce consistent changes in circulating miR-122. The predominance of the Omicron variant during our study period, in contrast to the above studies conducted during the first waves of the pandemic, may partly explain this result, given its association with milder disease and, consequently, less pronounced systemic inflammation and liver involvement.

### 3.7. miR-122 Expression and Demographics

In our study, baseline miR-122 expression showed a modest inverse relationship with age, which appeared sensitive to adjustment for other factors. A similar pattern was described by Devaux et al. in a cohort of patients with out-of-hospital cardiac arrest [38]. By contrast, in COVID-19 patients, Gutmann et al. reported a stronger inverse correlation with age that persisted after adjustment [6].

No significant differences in miR-122 expression were observed between sexes in our cohort, which was expected given that the *MIR122* gene is located on chromosome 18 [39] rather than on the X chromosome, as is the case for other microRNAs.

### 3.8. miR-122 Expression and Body Mass Index

A positive association between miR-122 and BMI has frequently been reported, although the independent nature of this relationship has not been demonstrated [24,40]. In our cohort of COVID-19 patients, no significant correlation was observed between miR-122 expression and BMI.

### 3.9. miR-122 Expression in Chronic Kidney Disease

It should be noted that patients with chronic kidney disease (CKD) may exhibit altered circulating miR-122 levels, which is why an estimated glomerular filtration rate (eGFR) below 50 mL/min/1.73 m^2^ was an exclusion criterion in our study. On the one hand, increased miR-122 levels have been reported in patients with early-stage CKD, possibly reflecting alterations in lipid metabolism, in which miR-122 is involved [25,41]. Conversely, lower miR-122 levels have been described in patients with end-stage CKD, potentially explained by increased circulating RNase levels [42]. However, the circulating kinetics and clearance mechanisms of microRNAs are not yet fully understood, and further studies are needed to clarify these aspects [43].

### 3.10. Strengths and Limitations of the Study

An important strength of this study is the timeliness of the investigated cohort, which included patients enrolled during the period of Omicron variant circulation—a phase of the pandemic that has been scarcely represented in studies evaluating miR-122 in COVID-19. Moreover, our study used a longitudinal design, allowing dynamic assessment of miR-122 expression over the course of COVID-19 treatment and providing a temporal perspective that has been rarely addressed in the existing literature. To our knowledge, this is the first study to investigate miR-122 expression in relation to COVID-19 therapy, explicitly addressing potential hepatocellular injury associated with its administration. Furthermore, the data derive from a real-world setting, reflecting medication exposures encountered in current practice, including polypharmacy, thereby enhancing the clinical relevance of our findings. An additional strength of the study is the comprehensive analysis of the relationship between miR-122 and a broad range of clinical, biochemical, and inflammatory parameters, allowing for an integrated and biologically coherent interpretation of the results.

This study is subject to limitations related to its observational design and modest sample size, highlighting the need for larger future studies. In addition, absolute quantification of circulating miR-122 levels and longer follow-up with multiple measurements, including in untreated COVID-19 patients and uninfected participants, could provide further insights into miR-122 dynamics and its relationship with administered treatments.

## 4. Materials and Methods

### 4.1. Study Design and Participants

We conducted a prospective observational cohort study that included three groups of participants: (1) hospitalized patients with SARS-CoV-2 infection who received COVID-19–specific treatment, with two blood samples collected for longitudinal miR-122 assessment, at baseline (before or within 24 h of treatment initiation) and at follow-up (after 5 days of treatment); (2) patients with SARS-CoV-2 infection who did not receive COVID-19–specific treatment, and (3) non-COVID-19 participants, with single blood sample collected for miR-122 analysis.

In the first two groups, we included adult patients (≥18 years) with COVID-19 confirmed by either a positive polymerase chain reaction (PCR) test or a rapid antigen test for SARS-CoV-2, who were consecutively screened and enrolled after presentation to the Emergency Department or after admission to the “Prof. Dr. Matei Balș” National Institute for Infectious Diseases (MBNIID), Bucharest, Romania, between March 2022 and July 2023. The third group consisted of non-COVID-19 participants (e.g., employees of the MBNIID) selected to ensure representation across the age, sex, and BMI subgroups observed in patient groups 1 and 2. Non-COVID-19 participants were included for comparative analyses with COVID-19 patients and were not individually matched to cases.

Participants with pre-existing liver disease (including viral hepatitis, liver fibrosis, hepatocellular carcinoma, etc.), CKD with an eGFR below 50 mL/min/1.73 m^2^, chronic alcohol consumption, or exposure to medications with a high risk of hepatotoxicity (e.g., chemotherapy) were excluded. Patients who had initiated COVID-19–specific treatment more than 24 h prior to presentation were also excluded.

Patients received nationally recommended treatment for SARS-CoV-2 infection according to disease severity, drug availability, and the clinical judgment of the attending physician.

All enrolled participants provided written informed consent. The study was approved by the Bioethics Committee of the MBNIID (C02654/16 March 2022).

### 4.2. Blood Sample Collection, Preparation, and Storage

Blood samples collected for miR-122 analysis were drawn into BD Vacutainer EDTA tubes and processed within 30 min at room temperature or stored for up to 4 h at 4 °C prior to processing. Samples were subsequently centrifuged at 3700 revolutions per minute (rpm) for 10 min at 8 °C, and the resulting plasma was aliquoted and stored at −80 °C. Samples were thawed only once, at the time of analysis. miR-122 determination was performed at the “Ștefan Nicolau” Institute of Virology, Bucharest, Romania.

### 4.3. RNA Isolation and Complementary DNA Synthesis

Total RNA was isolated from plasma samples using TRIzol™ reagent (Invitrogen™, Thermo Fisher Scientific Inc., Waltham, MA, USA) and subsequently purified with the RNeasy Mini Kit (Qiagen, Hilden, Germany) according to the manufacturers’ instructions. RNA quantity and integrity were determined using an Experion analyzer (Bio-Rad, Berkeley, CA, USA), and only samples with an A260/280 ratio > 1.8 and an RNA Quality Indicator (RQI) > 7 were included in further analyses. For complementary DNA (cDNA) synthesis, 1 µg of total RNA was reverse transcribed using the High-Capacity cDNA Reverse Transcription Kit (Thermo Fisher Scientific Inc., Waltham, MA, USA) following the manufacturer’s protocol.

### 4.4. Quantitative Real-Time PCR

Circulating miR-122 expression levels were measured by quantitative real-time PCR (qPCR) using Maxima SYBR Green/ROX qPCR Master Mix (Thermo Fisher Scientific Inc., Waltham, MA, USA) and on ABI 7300 Real Time System (Applied Biosystems, Thermo Fisher Scientific Inc., Waltham, MA, USA) following manufacturer’s instructions. Each biological sample was run in duplicate and U6 small nuclear RNA was used as an endogenous control for normalization. Relative miR-122 expression was calculated as log_10_(2^−ΔCt^), where ΔCt represents the difference between miR-122 and U6 cycle threshold (Ct) values. The following primer sequences were used for miR-122: forward 5′-GTGACAATGGTGGAATGTGG-3′ and reverse 5′-AAAGCAAACGATGCCAAGAC-3′ [44]; and for U6: forward 5′-CTCGCTTCGGCAGCACATATACT-3′ and reverse 5′-ACGCTTCACGAATTTGCGTGTC-3′ [45].

### 4.5. Data Collection

We entered enrolled patients’ data into a Microsoft Office Excel database, including demographics, comorbidities, BMI, COVID-19 immunization, severity of COVID-19, treatment received for COVID-19 or comorbidities, and laboratory data. For hospitalized patients, a broader panel of blood tests was available than for outpatient participants, including inflammatory markers such as IL-1, IL-6, TNF-α, and PAI-1. For group 1, results from two sequential blood samples were recorded, allowing longitudinal assessment of miR-122 alongside other laboratory parameters.

### 4.6. COVID-19 Severity Classification

Pulmonary involvement was assessed in all patients by chest X-ray and/or chest computed tomography, according to clinical indication. COVID-19 severity was classified as follows: (1) mild, in patients presenting with symptoms of an acute upper respiratory tract infection without pulmonary involvement; (2) moderate, in patients with pulmonary involvement but not requiring supplemental oxygen; and (3) severe, in patients with pulmonary involvement who required oxygen therapy.

### 4.7. Statistical Analysis

Statistical analysis of patient data was performed using IBM^®^ SPSS^®^ Statistics, Version 23.0, New York, NY, USA (released 2015). For the quantitative variables, we presented the median and interquartile range (IQR) and for the nominal and ordinal variables the frequencies. Comparisons across multiple groups were performed using the Kruskal–Wallis test, while pairwise group comparisons were carried out with the Mann–Whitney U test, applying Bonferroni correction for multiple testing. Fisher’s exact test was used for dichotomous variables. Associations between variables were assessed using Spearman’s rank correlation. For patients with two sequential blood measurements, paired comparisons between baseline and follow-up values were performed using the Wilcoxon signed-rank test. Furthermore, multivariate linear regression analyses were performed to determine which variables were independently associated with miR-122 expression. *p* < 0.05 was set for statistical significance.

## 5. Conclusions

In conclusion, we found no significant associations between miR-122 relative expression and remdesivir administered for standard treatment durations (3–5 days) or other COVID-19–specific therapies. However, further investigation of miR-122 dynamics in patients receiving prolonged courses of remdesivir is justified in order to clarify a potential duration-dependent effect. In contrast, therapeutic doses of paracetamol may be associated with liver function impairment in COVID-19 patients, likely through cumulative effects with other disease-related factors. In addition, our findings support an inverse relationship between miR-122 expression and systemic inflammation, suggesting that reduced circulating miR-122 levels may reflect adaptive hepatic responses to inflammatory stress. While not definitive, these results provide relevant preliminary insights into the complex interplay between treatment exposure, systemic inflammation, and hepatic responses in COVID-19, and underscore the need for larger, systematic studies.

## Figures and Tables

**Table 1:** COVID-19 and related complications therapy administered during hospitalization.

Treatment	Treated Patients (*n* = 57)
LMWH, *n* (%)	41 (71.9%)
Remdesivir, *n* (%)	55 (96.5%)
Treatment duration (days), median (IQR)	5 (4–5)
Treatment duration ≥ 5 days, *n* (%)	32 (56.1%)
Favipiravir, *n* (%)	1 (1.8%)
Treatment duration (days)	5
Molnupiravir, *n* (%)	2 (3.5%)
Treatment duration (days), median (IQR)	3.5 (3–4)
Tocilizumab, *n* (%)	1 (1.8%)
No. of administrations	2
Cumulative dose (mg)	1600
Monoclonal antibody therapies against SARS-CoV-2, *n* (%)	2 (3.5%)
Systemic corticosteroids, *n* (%)	13 (22.8%)
Treatment duration (days), median (IQR)	5 (4–6)
Cumulative dose (dexamethasone-equivalent mg ^a^), median (IQR)	36 (16–36)
Antibiotics, *n* (%)	19 (33.3%)
Beta-lactams	17 (29.8%)
Linezolid	3 (5.3%)
Doxycycline	2 (3.5%)
Macrolides	1 (1.8%)
Treatment duration (days), median (IQR)	5 (4–5.5)

^a^ Most patients received dexamethasone (*n* = 10). When other corticosteroids were administered, doses were converted to dexamethasone-equivalent values. IQR, interquartile range; LMWH, low molecular weight heparin.

**Table 2:** Correlations of baseline miR-122 with clinical and laboratory parameters in COVID-19 patients.

Variable	All (*n* = 78)		≥5 Days of COVID-19 Symptoms (*n* = 41)	
	Spearman’s ρ	*p* Value	Spearman’s ρ	*p* Value
**Clinical parameters**				
Age	−0.278	0.014 *	−0.369	0.018 *
Charlson comorbidity index	−0.287	0.011 *	−0.380	0.014 *
Cardiovascular diseases	−0.189	0.066	−0.425	0.006 *
Sartans	−0.264	0.019 *	−0.473	0.002 *
SpO2	0.293	0.010 *	0.292	0.064
COVID-19 severity	−0.229	0.043 *	−0.323	0.039 *
NSAIDs prior presentation	0.118	0.252	0.379	0.015 *
**Laboratory parameters**				
Thrombocytes	0.234	0.041 *	0.376	0.017 *
Lymphocytes	0.399	<0.001 *	0.409	0.009 *
Fibrinogen	−0.296	0.012 *	−0.332	0.045 *
CRP	−0.248	0.030 *	−0.355	0.025 *
IL-6	−0.359	0.020 *	−0.407	0.054
Urea	−0.168	0.103	−0.406	0.008 *
Creatinine	0.054	0.609	−0.031	0.849

CRP, C-reactive protein; IL-6, interleukin-6; NSAIDs, nonsteroidal anti-inflammatory drugs; SpO_2_, peripheral oxygen saturation. * *p* < 0.05.

## Data Availability

The original contributions presented in this study are included in the article/Supplementary Material. Further inquires can be directed to the corresponding authors.

## Supplementary Materials

The following supporting information can be downloaded at: https://www.mdpi.com/article/10.3390/ijms27052288/s1.
