# Supplementary material for: Circulating Liver-Enriched miR-122 in COVID-19 Patients: A Longitudinal Real-Life Study
Source: Int J Mol Sci. 2026 Feb 28;27(5):2288. doi: 10.3390/ijms27052288 (PMC12984953; doi:10.3390/ijms27052288)
Supplement: Supplementary file 1 [file ijms-27-02288-s001.zip › ijms-4130420-supplementary.pdf]

Table S1. Baseline characteristics of the study participants.

Variable	Non-COVID-19 participants (1)	Mild COVID-19 (2)	Moderate COVID-19 (3)	Severe COVID-19 (4)	Global *p* value[a]	Significant between-group differences[b]
**Demographics and clinical data**						
N (%)	18 (18.8%)	38 (39.6%)	32 (33.3%)	8 (8.3%)	-	-
Age (years)	51 (44-58)	54.5 (39-67)	66 (52.5-75.5)	77 (72-80.5)	< 0.001*	1 vs 3, 1 vs 4, 2 vs 4
Female sex [c]	12 (66.7%)	30 (78.9%)	21 (65.6%)	4 (50%)	0.327	-
Charlson Comorbidity Index	0.5 (0-1)	1 (0-3)	3 (1-4)	4 (4-5.5)	< 0.001*	1 vs 3, 1 vs 4, 2 vs 4
BMI (kg/m^2)	28 (24.8-31)	25 (22-30)	27.25 (22.75-28)	24.75 (21.5-32)	0.448	-
No. of COVID-19 vaccine doses	NA	2 (2-3)	2 (0-3)	0 (0-1)	0.035*	2 vs 4
Days since symptom onset	-	4 (3-5)	5 (4-7)	5 (4-5.5)	0.321	-
SpO2 at presentation (%)	-	98 (96-98)	97 (96-98)	89.5 (86-91)	< 0.001*	2 vs 4, 3 vs 4
**Inflammatory biomarkers**						
Neutrophil count (cel/µL)	2,270 (2,050-2,600)	3,000 (1,700-5,470)	3,455 (1,885-6,650)	3,970 (3,350-7,320)	0.034*	1 vs 4
Lymphocyte count (cel/µL)	4,555 (3,560-5,690)	2,005 (940-2,700)	1,500 (855-3,065)	830 (740-1,135)	< 0.001*	1 vs 2, 1 vs 3, 1 vs 4
Fibrinogen (mg/dL)	318 (261-357)	371 (264-458.5)	416.5 (297.5-519.5)	515 (373-607.5)	0.031*	-
CRP (mg/L)	2.05 (0-12.2)	9.595 (4.3-29.2)	8.6 (3.2-16.7)	62.5 (29.1-149.5)	0.003*	1 vs 4, 2 vs 4, 3 vs 4
Ferritin (ng/mL)	NA	50.3 (31.9-93.4)	69.9 (41.4-155.5)	368.7 (21.1-484.3)	0.374	-
IL-1 (pg/mL)	NA	3.6 (1.1-20.3)	2.95 (0.5-23.8)	15.8 (4.45-30.55)	0.383	-
IL-6 (pg/mL)	NA	150 (21-634.7)	683.2 (67.7-1,220)	2,460.3 (787.8-3,381)	0.029*	2 vs 4
TNF-α (pg/mL)	NA	29.9 (16.15-107.3)	158.69 (61.95-231.25)	244.5 (94.95-614.2)	0.046*	-
PAI-1 (ng/mL)	NA	255.4 (225.5-400.3)	353 (318.8-502)	336.6 (220.4-438.8)	0.530	-
**Hematology and coagulation**						
Hemoglobin (g/dL)	13.85 (12.9-14.6)	13.4 (12.4-13.8)	12.8 (12.2-13.75)	12.7 (11.7-14.35)	0.232	-
Platelet count (×10^3 cel/µL)	285.5 (270-304)	241 (203-293)	248 (190.5-293.5)	157 (140-231.5)	0.008*	1 vs 4
INR	0.935 (0.9-0.97)	0.995 (0.96-1.04)	1.01 (0.93-1.07)	1.065 (1-1.26)	0.002*	1 vs 2, 1 vs 3, 1 vs 4
D-dimers (µg/mL)	NA	0.319 (0.171-0.424)	0.402 (0.325-0.605)	0.487 (0.325-2.189)	0.035*	-

**Liver biomarkers**						
ALT (U/L)	35 (27-40)	29.75 (24.5-47)	28 (23.75-34.25)	29.25 (27.5-32)	0.707	-
AST (U/L)	32.75 (25.5-36)	36.75 (28.5-47)	34.5 (29.25-44.5)	42.25 (32-57)	0.189	-
GGT (U/L)	25.5 (23.5-40.5)	23.5 (19.25-39.5)	25.5 (20-40.25)	26.5 (20.75-53)	0.895	-
ALP (U/L)	62 (53-81)	68.5 (59-91)	64 (50-73)	56 (53.5-70)	0.350	-
TBIL (mg/dL)	0.6 (0.5-0.8)	0.6 (0.35-0.75)	0.6 (0.5-0.75)	0.9 (0.6-0.95)	0.435	-
LDH (U/L)	196 (190-202)	188.5 (175-216)	200 (167-235)	261 (228-284)	0.067	-
Albumin (g/dL)	NA	4.5 (4.45-4.6)	4.4 (4-4.7)	3.65 (3.1-4.1)	0.021*	2 vs 4
miR-122	- 2.05 (-2.95; -1.23)	-1.71 (-2.20; -0.86)	-1.91 (-2.94; -1.50)	-2.25 (-2.44; -2.14)	0.291	-
**Renal and metabolic biomarkers**						
Urea (mg/dL)	36.5 (27-40)	29 (22-36)	29.5 (27-39.5)	32 (27.5-47.5)	0.265	-
Creatinine (mg/dL)	0.7 (0.6-0.9)	0.8 (0.7-1.1)	0.7 (0.6-0.9)	0.75 (0.6-1.05)	0.453	-
Lipase (mg/dL)	75.5 (50-132)	79 (58-119)	87.5 (53-153.5)	164 (107-168)	0.511	-
Glucose (mg/dL)	100 (90-108)	105 (98-126)	101 (97.5-116)	102 (91.5-120)	0.440	-
**Cardiac biomarkers**						
Troponin (ng/mL)	NA	0.03 (0.03-0.03)	0.03 (0.03-0.03)	0.03 (0.03-1.649)	0.071	-
CK (U/L)	90 (71.5-113)	64 (51-81)	74 (46-119)	108.5 (56-294.5)	0.207	-
CK-MB (U/L)	14 (14-16.5)	11 (7-16)	12 (9-17)	10 (4.5-14.5)	0.254	-

[a] Kruskal–Wallis test. [b] Pairwise group comparisons using the Mann–Whitney U test with Bonferroni correction for multiple testing; significant pairwise differences are indicated as group numbers (e.g., 1 vs 3). [c] Fisher's exact test. * Statistically significant. Data are presented as number (%) or median (IQR). ALP, alkaline phosphatase; ALT, alanine aminotransferase; AST, aspartate aminotransferase; BMI, body mass index; CK, creatine kinase; CK-MB, creatine kinase–MB; CRP, C-reactive protein; GGT, gamma-glutamyl transferase; IL-1, interleukin-1; IL-6, interleukin-6; INR, international normalized ratio; IQR, interquartile range; LDH, lactate dehydrogenase; NA, not available; PAI-1, plasminogen activator inhibitor-1; SpO_2, peripheral oxygen saturation; TBIL, total bilirubin; TNF-α, tumor necrosis factor-α.

Table S2. Distribution and severity of abnormalities in liver-related biochemical parameters.

Variables	At presentation	At follow-up[a]	Total[b]
**COVID-19 participants with two time-point measurements (*n* = 57)**			
Elevated aminotransferases (ALT and/or AST), ***n*** (%)	14 (24.6%)	18 (31.6%)	22 (38.6%)
Elevated ALT, ***n*** (%)	10 (17.5%)	17 (29.8%)	19 (33.3%)
Grade 1: (1–3) × ULN	8 (14%)	16 (28%)	17 (29.8%)
Grade 2: [3–5) × ULN	2 (3.5%)	1 (1.8%)	2 (3.5%)
Elevated AST, ***n*** (%)	13 (22.8%)	11 (19.3%)	19 (33.3%)
Grade 1: (1–3) × ULN	13 (22.8%)	11 (19.3%)	19 (33.3%)
Elevated GGT, ***n*** (%)	7 (12.3%)	11 (19.3%)	11 (19.3%)
Grade 1: (1–3) × ULN	6 (10.5%)	10 (17.5%)	10 (17.5%)
Grade 2: [3–5) × ULN	1 (1.8%)	1 (1.8%)	1 (1.8%)

Elevated ALP, ***n*** (%)	1 (1.8%)	0	1 (1.8%)
Elevated LDH, ***n*** (%)	16 (28%)	8 (14%)	19 (33.3%)
Elevated TBIL, ***n*** (%)	2 (3.5%)	2 (3.5%)	4 (7%)
**COVID-19 participants with a single measurement (*n* = 21)**			
Elevated aminotransferases (ALT and/or AST), ***n*** (%)	3 (14.3%)	-	-
Elevated ALT, ***n*** (%)<br>Grade 1: (1–3) × ULN<br>Grade 2: [3–5) × ULN<br>Grade 3: ≥5 × ULN	3 (14.3%)<br>2 (9.5%)<br>0<br>1 (4.8%)	-	-
Elevated AST, ***n*** (%)<br>Grade 1: (1–2) × ULN	1 (4.8%)<br>1 (4.8%)	-	-
Elevated GGT, ***n*** (%)<br>Grade 1: (1–3) × ULN	2 (9.5%)<br>2 (9.5%)	-	-
Elevated ALP, ***n*** (%)	0	-	-
Elevated LDH, ***n*** (%)	0	-	-
Elevated TBIL, ***n*** (%)	0	-	-
**Non-COVID-19 participants (*n* = 18)**			
Elevated ALT, ***n*** (%)<br>Grade 1: (1–2) × ULN	1 (5.6%)<br>1 (5.6%)	-	-
Elevated AST, ***n*** (%)	0	-	-
Elevated GGT, ***n*** (%)<br>Grade 1: (1–2) × ULN	1 (5.6%)<br>1 (5.6%)	-	-
Elevated ALP, ***n*** (%)	0	-	-
Elevated LDH, ***n*** (%)	0	-	-
Elevated TBIL, ***n*** (%)	0	-	-

[a] Around day 5 of treatment. [b] At presentation and/or at follow-up. ULN, upper limit of normal.

Table S3. Distribution of major chronic medications among study participants.

**Treatment**	**COVID-19 patients (Overall, *n* = 78)**	**COVID-19 patients with longitudinal assessment (*n* = 57)**	**Non-COVID-19 participants (*n* = 18)**
Beta-blockers	24 (30.8%)	23 (40.4%)	2 (11.1%)
Statins	22 (28.2%)	20 (35.1%)	1 (5.6%)
Diuretics	20 (25.6%)	18 (31.6%)	3 (16.7%)
Angiotensin-converting enzyme inhibitors	19 (24.4%)	15 (26.3%)	4 (22.2%)
Angiotensin receptor blockers	14 (17.9%)	12 (21.1%)	0
Metformin	11 (14.1%)	9 (15.8%)	1 (5.6%)
Calcium channel blockers	8 (10.3%)	7 (12.3%)	2 (11.1%)
Proton pump inhibitors	6 (7.7%)	6 (10.5%)	1 (5.6%)
Oral anticoagulants	6 (7.7%)	6 (10.5%)	0
Clopidogrel	3 (3.8%)	3 (5.3%)	0
Total	58 (74.4%)	47 (82.5%)	5 (27.8%)

Data are presented as *n* (%).

Table S4. Changes in laboratory parameters between baseline and follow-up in COVID-19 patients (*n* = 57).

Variable	Baseline, median (IQR)	Follow-up, median (IQR)	Direction of change	p value (Wilcoxon)
Lymphocytes (cel/μL)	1,210 (790-2,010)	2,150 (1,620-2,350)	↑ increase	< 0.001
Thrombocytes (×10^3 cel/μL)	229.5 (172-278)	275 (205-328)	↑ increase	< 0.001
INR	1.01 (0.94-1.1)	1.06 (1.03-1.15)	↑ increase	0.018
D-dimers (μg/mL)	0.407 (0.308-0.611)	0.270 (0.193-0.413)	↓ decrease	0.007
Fibrinogen (mg/dL)	408.5 (309-534)	284 (257-362)	↓ decrease	< 0.001
CRP (mg/L)	11.1 (4.09-41.3)	9.1 (2.2-20.5)	↓ decrease	0.001
